# Durable remission of refractory and advanced stage mycosis fungoides/sezary syndrome utilizing an “outpatient” alemtuzumab, fludarabine-based reduced intensity allogeneic hematopoietic cell transplantation

**DOI:** 10.1038/s41409-024-02380-6

**Published:** 2024-09-09

**Authors:** Phuong Vo, Ramaprasad Srinivasan, Enkhtsetseg Purev, Emily McDuffee, Tatyana Worthy, Reem Shalabi, Kristen Wood, Brian Wells, Robert Reger, David Stroncek, Nancy Geller, Georg Aue, Xin Tian, Richard Childs

**Affiliations:** 1 National Heart, Lung and Blood Institute, National Institutes of Health, Bethesda, MD USA; 2 National Cancer Institute, National Institutes of Health, Bethesda, MD USA; 3 Department of Transfusion Medicine, National Institutes of Health, Bethesda, MD USA; 4 Office of Biostatistics Research, National Heart, Lung and Blood Institute, National Institutes of Health, Bethesda, MD USA; 5 Present Address: Division of Clinical Research, Fred Hutchinson Cancer Research Center, Seattle, WA USA; 6 Present Address: Department of Medicine, University of Washington, Seattle, WA USA

**Keywords:** T-cell lymphoma, Stem-cell research

## To the Editor:

Mycosis fungoides (MF) and its leukemic variant Sezary syndrome (SS), the most prevalent subtypes of primary cutaneous T-cells lymphomas (CTCL), present significant treatment challenges in advanced stages. Currently, there is no treatment available for subjects with advanced disease that improves overall survival. Allogeneic hematopoietic stem cell transplantation (HCT) had been utilized for the treatment of MF/SS [1–3]. Unfortunately, a 20% or greater risk of transplant related mortality (TRM) precludes the use of conventional “dose-intensive” allogeneic HCT in many patients. Data have shown the potential TRM risk associated with conditioning can be mitigated, to some extent, by utilizing a reduced-intensity conditioning (RIC) [4]. In this study, we describe a novel outpatient-based RIC utilizing fludarabine and alemtuzumab. Alemtuzumab is a monoclonal antibody targeting CD52, a glycoprotein expressed on most MF cells and over 95% of peripheral blood lymphocytes, monocytes and macrophages [5]. We theorized that this RIC regimen would provide the essential tumor-debulking and facilitate the necessary donor T-cell chimerism levels to induce curative GVT effects in patients with advanced forms of MF/SS.

Between February 2004 and May 2014, 5 subjects (median age of 57, ranged 27–59 years at the time of HCT) with relapsed advanced disease of stages III (1 patient) or IV (4 patients) MF/SS underwent RIC with alemtuzumab and fludarabine followed by PBSC transplantation at NIH Clinical Center. All patients had failed a median 3 prior treatment regimens (range 2–7) for MF/SS. All subjects consented in writing to participate in this NHLBI Institutional Review Board approved protocol (Clinical trials.gov: NCT00047060).

The conditioning regimen consisted of alemtuzumab 3 mg on day −28, 10 mg on day −27, and 30 mg on day −26, −24, −22, −19, −17, −15 (total of 193 mg), followed by fludarabine 25 mg/m^2^/day on days −5 to −1 (total of 125 gm/m^2^). GVHD prophylaxis consisted of cyclosporine A alone (CSA) beginning on day −4. On day 0, unmanipulated G-CSF mobilized PBSC allograft obtained from an HLA-identical relative was infused. Patients with disease progression and/or low degrees of donor T-cell chimerism (<50% donor) received escalating doses of donor lymphocyte infusions (DLI) (5 × 10^6^/kg; 1 × 10^7^/kg; 5 × 10^7^/kg) with a goal to convert T-cell chimerism from mixed to full donor.

**Fig. 1:**
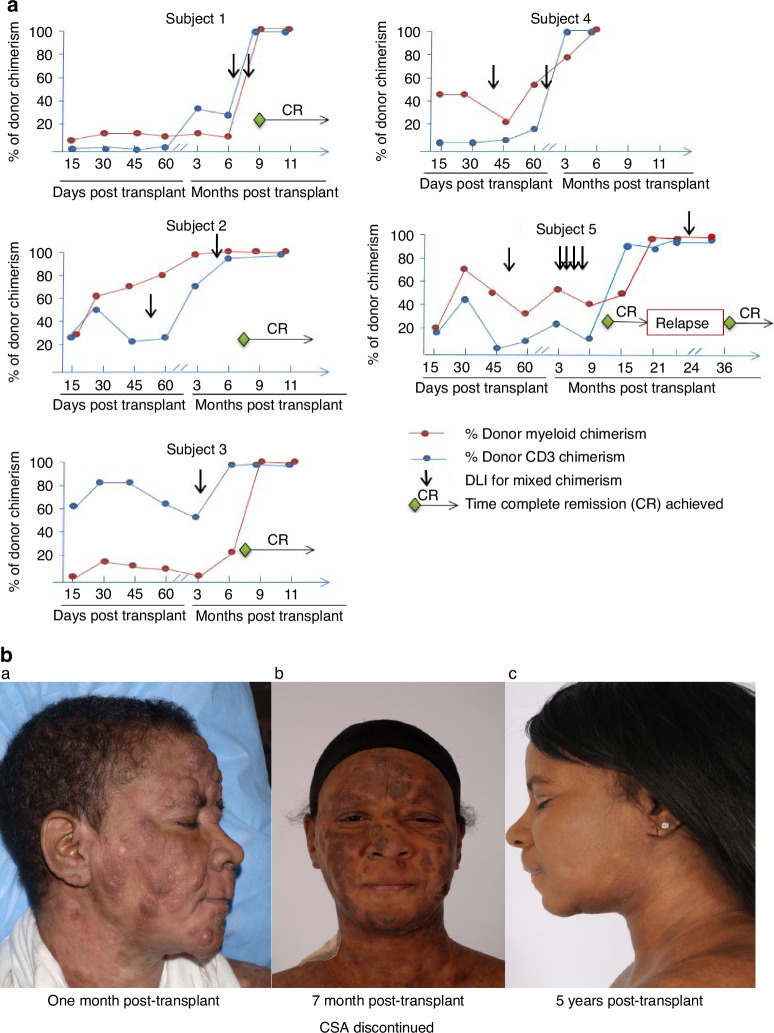
Lineage specific chimerism and patient’s clinical response. **a** Lineage specific chimerism of each subject. T-cell and myeloid chimerism were determined by PCR analysis of short tandem repeat (STR) on day 15, 30, 45, 60, 100, and continued monthly until full donor chimerism was achieved in both lineages. Complete donor chimerism was defined as >95% donor derived cells in peripheral blood in a specific lineage. **b** Patient #2 who achieved a long-lasting remission of stage 3 mycosis fungoides following alemtuzumab/Fludarabine based RIC allogeneic hematopoietic stem cell transplantation. Serial post-transplant photographs show (a) biopsy-confirmed MF tumor nodules that persisted 1 month after HCT (b) becoming hyperkeratotic, macular and smaller in size following CSA tapering at 7-month post HCT; (c) 5 years after HCT, the patient remained in remission with normal appearing skin without any evidence of cGVHD.

From the initiation of conditioning to the completion of donor engraftment, none of the subjects has experienced profound, prolonged neutropenia or thrombocytopenia. Only three of the five patients became neutropenic (ANC < 500/uL) and had rapid neutrophil recovery occurring after a median of 5 days (range 4–7 days). No patients required platelet transfusions. Median hospitalization duration was only 3 days pre/post-transplant and 11 days within 100 days of the post-transplant period, respectively. The main reasons for hospitalization were infections that required intravenous antimicrobials. All 5 subjects initially had mixed lympho-hematopoietic chimerism but eventually converted to full donor chimeras in both myeloid and T-cell lineages; full donor T-cell chimerism was achieved at a median 7 month post-transplant (range 4–22 months); full donor myeloid chimerism was achieved at median 9 months post-transplant (range 3–18, Fig. 1a). No patients developed acute GVHD prior to DLI. At day 100, the cumulative incidence of GVHD was 0. One subject developed grade 3 gut GVHD 54 days after his second DLI infusion given for low donor T-cell chimerism. Three subjects developed limited skin chronic GVHD that resolved with short course of steroids. Immunosuppressive therapy was discontinued in all subjects at the time of analysis.

Four (80%) subjects achieved complete remission at median 8 months post-transplant (range 7–10 months) documented by skin biopsy, lab tests and imaging. Long-term survival analysis showed 4 patients survived at 3 years with an overall survival rate of 80%. Four patients remained in remission at their last follow-up at 13, 12 (Fig. 1b), 3 and 4 years, respectively. Two patients died: one (with a heavy smoking history, in remission) from a secondary head and neck squamous cell carcinoma and the other from an infection related to treatment for gut GVHD.

Data from European Society for Blood and Marrow Transplantation (EBMT) provided a clear picture of the value of allogeneic HCT as a therapeutic strategy for high-risk patients with advanced-stage MF/SS [6, 7]. However, transplant related mortality rates in the range 25–35% substantially curbed enthusiasm for the use of myeloablative allogeneic transplantation for this disease. Shiratori et al. reported outcomes of 9 patients with MF/SS who received RIC HCT, showing a 3-year overall survival rate of 85.7% (95% CI, 33.4–97.9%) without non-relapse mortality during a median follow-up of 954 days post-HCT [4]. A recent allogeneic transplant regimen using RIC conditioning with total skin electron beam therapy/total nodal irradiation and anti‐thymocyte globulin showed less favorable outcomes, with a one-year TRM of 7%, only 53% of transplanted patients maintaining complete remission, and 47% experiencing disease relapse at a median follow-up of 2.25 years [8]. These data suggest that despite reductions in TRM associated with RIC, disease progression is problematic and remains a major cause of treatment failure.

In our study, we formulated a RIC regimen utilizing immunosuppressive agents that were tailored to prevent graft rejection while simultaneously inducing cytoreductive anti-tumor effects against MF. Alemtuzumab was incorporated into the regimen because of its ability to induce significant and prolonged host lymphopenia, as well as in-vivo T-cell depletion of the allograft, ultimately facilitating donor engraftment without rapid donor T cell engraftment, which is associated with an increased risk of GVHD [9]. To prevent excessive depletion of T-cells in the allograft and potential graft rejection, alemtuzumab infusions were given from day −28 to day −15 as part of the conditioning regimen. Alemtuzumab was also selected to be part of the conditioning regimen as it is highly active against MF/SS with single agent response rates of up to 55% [10, 11]. Ensuring early disease control is important as GVT effects are often delayed for months following transplantation, particularly in patients with mixed donor T-cell chimerism.

The regimen was highly tolerable, preventing prolonged/profound neutropenia and thrombocytopenia, resulting in patients being hospitalized for an average of only 11 days by day 100 post-transplant. This represents substantial decrease in hospital stay when compared to conventional non-myeloablative stem cell transplant regimens which require hospital admission for an average 5–10 inpatient days during conditioning and 79 days following transplantation [12]. Most remarkably, in our pilot trial, 4/5 transplanted patients (80%) achieved a complete remission by a median 8 months post-transplant with 4 patients remaining in long-term remission at a median 4 years of follow-up (range, 38 months to 13 years). DLI infusions appeared essential to achieving remissions with this regimen, as CRs only occurred following 1 or more (median 2) DLI infusions. One subject (20%) relapsed 7 months after first CR and achieved 2nd CR following a DLI and treatment with brentuximab. The incorporation of alemtuzumab, an agent that is highly active against MF, into the conditioning regimen may have played an important role in preventing disease relapse following RIC allo-HCT.

Our data from this pilot trial suggests that a novel alemtuzumab and fludarabine-based conditioning approach could effectively induce lasting and possibly curative GVT effects post allogeneic HCT, with lower hematologic toxicity and reduced hospitalization days compared to conventional RIC conditioning, warranting further investigation through a larger trial to validate these promising results.

## Data Availability

Data are available on request to the corresponding author.
